# Research on Electric Field Homogenization in Radial Multi-Nozzle Electrospinning

**DOI:** 10.3390/nano14141199

**Published:** 2024-07-14

**Authors:** Jian Liu, Shoujun Dong, Chenghao Wang, Yanbo Liu, Shanshan Pan, Zhaosong Yin

**Affiliations:** 1 School of Mechanical Engineering, Tiangong University, Tianjin 300387, China; 2231050764@tiangong.edu.cn (S.D.); 2231050762@tiangong.edu.cn (C.W.); 2331050787@tiangong.edu.cn (S.P.); 2331050798@tiangong.edu.cn (Z.Y.); 2 School of Textile Science and Engineering, Wuhan Textile University, Wuhan 430200, China

**Keywords:** electrospinning, electric field homogenization, radial multi-nozzle, multiple jets, relative orientation of the vanes, numerical simulation

## Abstract

Electrospinning is an effective method to prepare nanofibers at present. Aiming at problems such as low spinnable viscosity and the low productivity of the traditional multi-needle, a radial nozzle was proposed in this paper. In order to solve the problem of end effects in multi-nozzle electrospinning, COMSOL Multiphysics 6.0 software was used to simulate the electric field in electrospinning with seven radial nozzles. And the influence on the electric field intensity and distribution of the structural parameters of the radial nozzle, including the number, length, tip-shape, and tip-pointing direction of the vanes, were studied. Then, the electric field intensity of any point on the central axis of a radial nozzle was obtained based on the principle of electric field superposition, and then the rotation angle of the vanes corresponding to the minimum Coulomb repulsion force on the target point was deduced. At last, the method of electric field homogenization of a rotating vane arrangement was obtained. In the simulation, the strength and homogenization of the electric field were taken as the research objective, and the optimum structure parameters of the radial nozzle were obtained; the uniform theory of the electric field based on the orientation of the vanes was verified. Then, electrospinning with seven radial nozzles was performed, and it was found that each radial nozzle can produce multiple jets during electrospinning, and the prepared electrospun membranes have even thickness and high porosity. What is more, the fibers are relatively finer and more uniform.

## 1. Introduction

Electrospinning is an effective method to prepare nanofibers by stretching a polymer solution under the electric field generated by a high-voltage DC power supply [1,2], and it has been widely studied and applied in many fields at present, such as environmental protection [3], biomedical science [4,5], aerospace [6,7], energy [8], micro–nano devices [9], and so on. With the continuous development of science and technology, multi-needle [10,11], porous tube [12,13], and needleless electrospinning methods [14,15,16,17,18,19,20] have emerged in recent decades. Among the many electrospinning technologies, traditional capillary needle electrospinning has many advantages, including low energy consumption, controllable liquid supply, and preparation of finer nanofibers, due to its tip-effect and closed liquid supply [21,22]. However, the problems, for example, low spinnable viscosity and low productivity, limit its further development [23,24].

**Figure 1:**
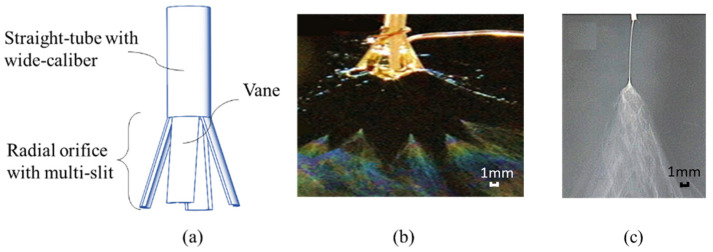
Comparison of the radial nozzle and capillary needle in electrospinning. (**a**) Structure of the radial nozzle. (**b**) Multiple jets in radial nozzle electrospinning. (**c**) Electrospinning with capillary needle.

In order to widen the viscosity range of the spinnable solution and increase the spinning output of needle electrospinning, it is necessary to increase the inner diameter of the needle appropriately. However, with the addition to the inner diameter, the solution will drop easily under the action of gravity when it flows to the orifice of the needle, which is not conducive to the formation of the spinning jets. According to the principle of surface tension in fluid mechanics [25], the surface tension will be added to the higher contact areas, and it is beneficial to increase the holding time of the droplet. Therefore, our research group put forward the concept of a radial nozzle with multi-slots for the first time. The design idea is that the orifice of the cylindrical straight tube with a wide caliber is divided into several sheet-like structures, on average, which are opened out with a certain angle to form a multi-slot radial orifice. And each sheet is called a “vane”, which is then named as the radial nozzle in this paper. As shown in Figure 1, the contact areas between the solution and the outlet are increased by partitioning. The surface tension between the solution and the vanes is increased by increasing the contact areas. Therefore, the spinning fluid can extend a longer distance along the radial orifice and prolong the holding time of the droplet; thus, it can increase the surface area of the spinning liquid and improve the stability of the spinning liquid surface, which provides the basic guarantee for improving the output of the electrospinning. And it is found that electrospinning with the radial nozzle can produce multiple jets, as shown in Figure 1b, and the productivity is higher than the traditional capillary needle compared in Figure 1c.

In traditional multi-needle electrospinning technology, there is mutual interference in the electric field among the needles. According to the principle of electric field superposition, the field strength of intermediate needles is lower than that of the ends, that is, the so-called end effect [26], which affects the quality of the electrospun fibers. In multi-needle electrospinning technology, needle structure and arrangement have great influence on field strength and homogenization. In this paper, ultiphysics 6.0 software was used to simulate the electric field and the influence on the electric field intensity and distribution of the structural parameters of the radial nozzle, including the number, length, end shape, and tip-pointing direction of the vanes, which were studied. And we propose a theory regarding uniform electric fields when blades are oriented in multi-slot radial nozzle configurations. In the simulation, the strength and homogenization of the electric field were taken as the research objective, and at last, the optimum structure parameters of the radial nozzle were obtained, and the proposed theory was validated.

## 2. Method and Discussion

### 2.1. Establishing the Model of the Electrospinning System with Solidworks

**Figure 2:**
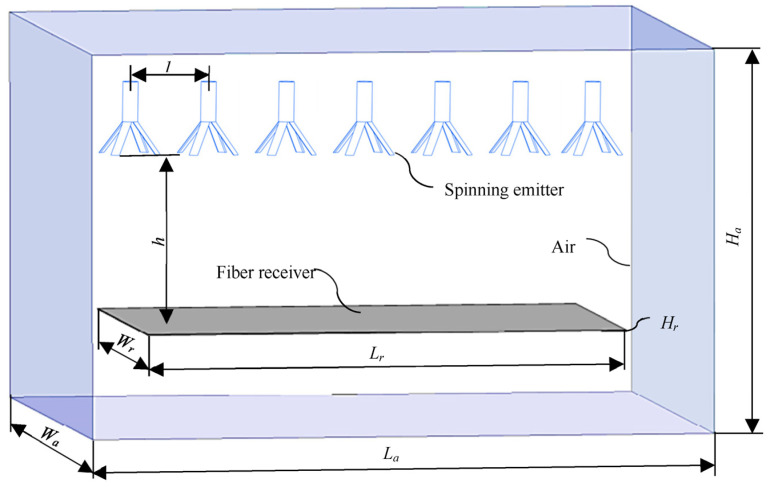
Model of the radial multi-nozzle electrospinning system.

Models of the electrospinning system generally include a spinning emitter, fiber receiver, and air. As shown in Figure 2, seven radial nozzles arranged linearly were selected as the spinning emitter, a plate was used as the fiber receiver, and a cuboid space contained the air. The electrospinning system model was established with Solidworks 2020 version software. The length of the straight tube in the radial nozzle is 4 mm, the inner diameter is 1.6 mm, the outer diameter is 2 mm, and the initial length of the vanes is 5 mm. Other parameters are shown in Table 1. Among them, *h* is the distance between the orifice of the radial nozzle and the upper surface of the fiber receiver, and *l* is the distance between the radial nozzles.

### 2.2. Electric Field Simulation of the Electrospinning System with COMSOL

**Figure 3:**
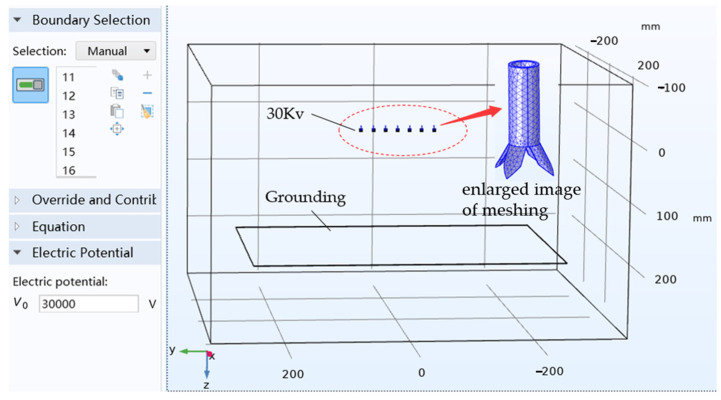
Electric field simulation of the radial multi-nozzle electrospinning system.

As shown in Figure 3, models with different parameters and arrangements are imported into the COMSOL Multiphysics 6.0 software, and the electric field of the radial multi-nozzle electrospinning system is simulated to obtain the optimized parameters with the most uniform and average electric field intensity. Firstly, the spinning emitter and fiber receiver are subtracted from the air in the simulation environment, and the steel properties are assigned to the emitter and receiver. Secondly, the spinning emitter is connected to 30 Kv, and the fiber receiver is grounded. Then, the model grid is set according to free tetrahedral mesh division. Finally, the electric field of the models is solved.

#### 2.2.1. Effects of the Number of Vanes on the Electric Field

**Figure 4:**
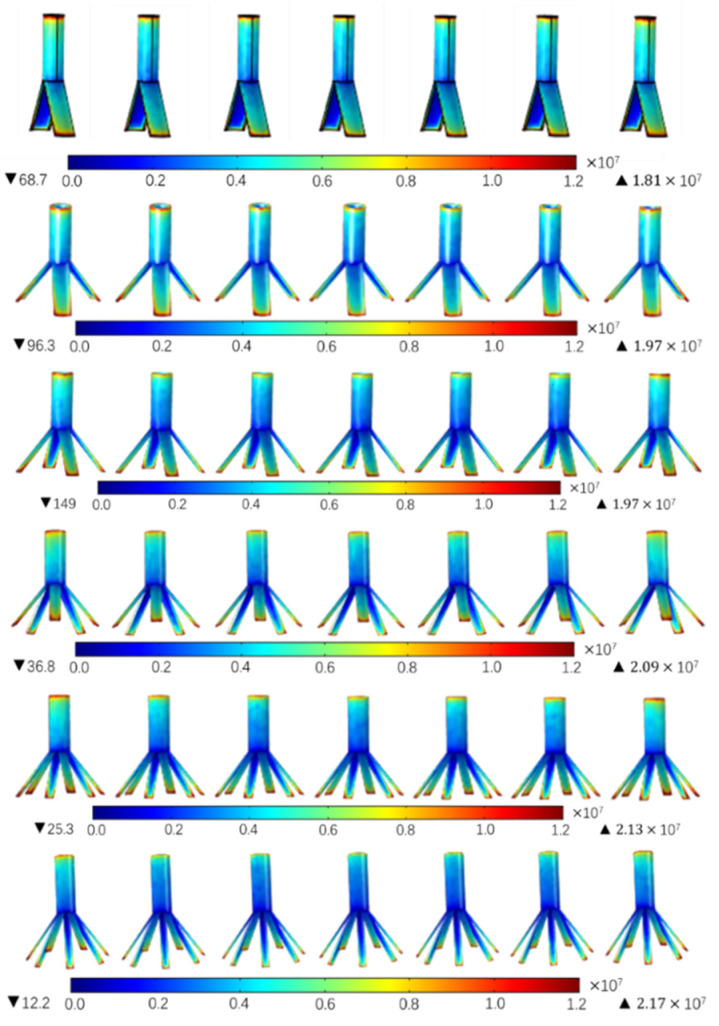
The distribution diagrams of the electric strength with different numbers of vanes.

Firstly, the effects of the number of radial vanes on the electric field were researched. The number of vanes chosen was 2, 3, 4, 5, 6, and 7. The electrostatic simulation results are shown in Figure 4.

We can see that the maximum field strength of the seven radial nozzles with two vanes is 1.81 × 10^7^ V/m. When the number of vanes is seven, the maximum field strength is 2.17 × 10^7^ V/m. And the regularity is obvious; that is, with the increase in the number of vanes, the maximum field strength of the electrospinning system model becomes larger and larger. The reasons are as follows: the radial shape of the radial nozzles is made by slicing the cylindrical straight tube, and if, regardless of the area loss caused by cutting, the total surface area of the radial nozzles with different numbers of vanes basically does not change, then when the same potential is applied to the nozzles, the total number of charges generated is the same basically. However the more vanes there are, the thinner the tips are, and because of the existence of the tip effect, the charges accumulate more easily. Thus, the maximum field strength becomes larger with the increasing number of vanes.

**Figure 5:**
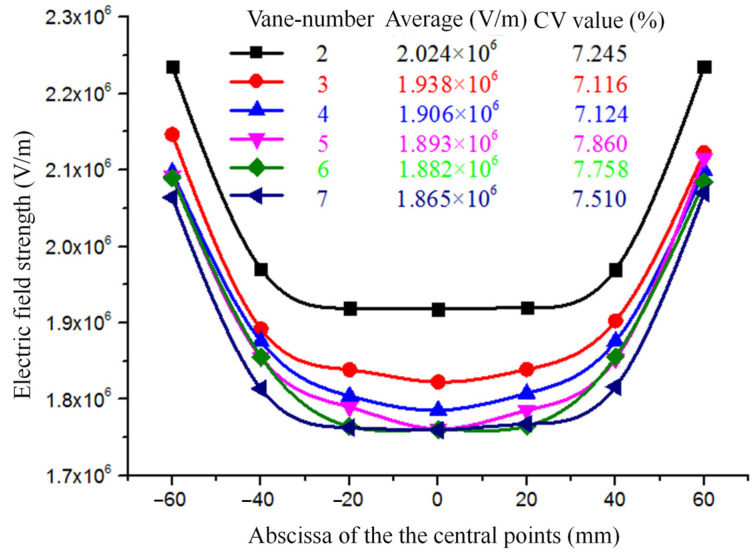
The field strength and distribution curve of the central points at the spinning-end surface of the seven nozzles with different numbers of vanes.

On the other hand, the positions of the Taylor-cone-inspired tips are usually located near the center of the radial nozzle [27,28]. Therefore, the field strength of the central points at the spinning end surface of the seven nozzles in the different electrospinning system models were derived with the COMSOL Multiphysics 6.0 software and the plane of the radial nozzle tips is defined as the “spinning-end surface”. At the same time, Origin 2021 version software was used to draw the field strength and distribution curve, as shown in Figure 5.

From Figure 5, it can be seen that the average field strength of the central points at the spinning-end surface of the radial nozzles decreased gradually and changed from 2.204 × 10^6^ V/m to 1.865 × 10^6^ V/m with the increase in the number of vanes. The Coulomb repulsion interactions among the vanes become more and more obvious with the increase in the number of vanes, which makes the electric field at the center point smaller and smaller. What is more, in the electrospinning process, the Coefficient of Variance, recorded as the CV value of the field strength, can reflect the uniformity of the electric field [29]. The smaller the CV value is, the more uniform the field strength is. Therefore, in the case that there is little difference in the average field strength, the CV value of the field strength should be considered emphatically. Thus the two, three, or four vanes with relatively small CV values for field strength are preferred. Although the average field strength of the seven nozzles with two vanes is the largest, their CV value is higher than that of the nozzles with three vanes and four vanes. In addition, the radial nozzle with two vanes is not adequately conductive to hold the droplets, and the spinning surface easily drops them. By comparison, the radial nozzle with four vanes can improve the holding time of the droplet in the spinning solution more effectively, so in the later section, taking the four-vane radial nozzle as an example and considering the length, the shape, and the pointing angle of the tips of the vanes, methods for improving the electric field intensity and uniformity of the radial nozzles were further studied to propose the final optimization scheme.

#### 2.2.2. Effects of the Length of the Vanes on the Electric Field

**Figure 6:**
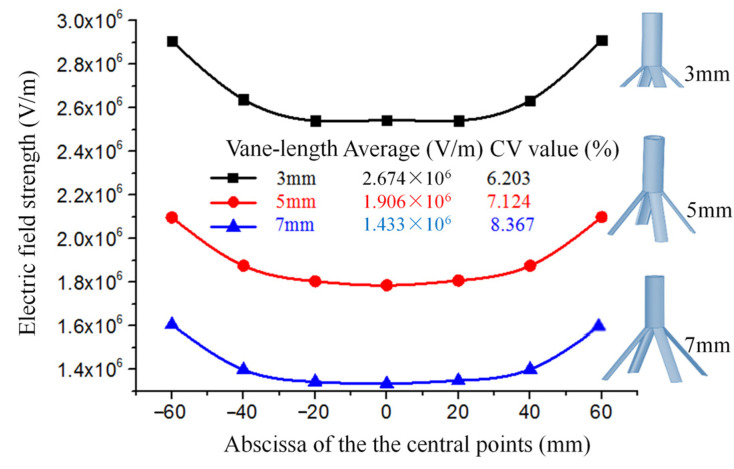
The field strength and distribution curve of the central points at the spinning-end surface of the seven nozzles with different lengths of vanes.

Aiming at the four-vane radial nozzle, the effects of the lengths of the vanes on the electric field were discussed. Firstly, three models of seven radial nozzles with linear arrangements were established. The lengths of the vanes were 3 mm, 5 mm, and 7 mm. The electrospinning system models were imported into the COMSOL Multiphysics 6.0 software to simulate the electric field. The simulation results such as the average field strength and CV values at the central points of the spinning-end surface of each electrospinning system are shown in Figure 6.

As shown in Figure 6, the average field strength of the central points at the spinning-end surface of the seven radial nozzles with lengths of 3 mm, 5 mm, and 7 mm are 2.674 × 10^6^ V/m, 1.906 × 10^6^ V/m, and 1.433 × 10^6^ V/m, respectively. This indicates that the longer the vanes are, the smaller the average field strength is. This depends mainly on the number of charges per unit area. Correspondingly, the CV values of the field strength are 6.203%, 7.124%, and 8.367% respectively. That is to say, the CV values of the field strength are becoming larger with the increase in the lengths of the vanes. It shows that the longer the vanes are, the greater the influence of the electric field among the nozzles is, and the more nonuniform the electric field is. Therefore, under the condition that the spinning solution can fully spread, we should choose the shorter vanes. And in this paper, the length of the vanes is determined to be 6 mm.

#### 2.2.3. Effects of the Tip Shape of the Vanes on the Electric Field

The produced electric field strength and distribution are different when applying the same voltage to conductors with different shapes. Especially if the conductor has tips, it is easier to collect charges and produce a higher field strength because of an existing tip effect [30]. Therefore, the end faces of the radial nozzle were designed in different shapes, preferably with tip shapes that include the rectangular, trapezoidal, polygonal, triangular, rhomboid, etc. In this section, we discuss what tip shape for the vanes is more conducive to reducing the energy consumption of the electrospinning.

**Figure 7:**
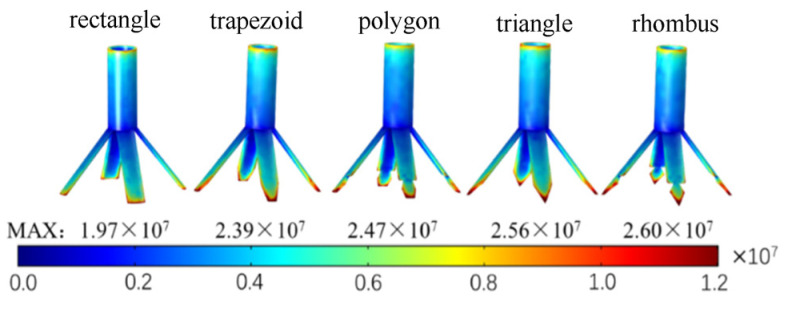
The electric field simulation of the electrospinning system models of seven radial nozzles with different tip shapes.

The electrospinning system models of the seven radial nozzles with different tip shapes were designed and imported into the COMSOL Multiphysics 6.0 software to simulate the electric field. The field strength nephogram of the intermediate nozzle and the maximum field strength of the electrospinning system are depicted in Figure 7.

Figure 7 shows that the maximum field strength increase from 1.97 × 10^7^ V/m to 2.6 × 10^7^ V/m in turn. The reason is that when the original rectangular end surface of the vane is processed into trapezoidal, polygonal (here, it is hexagonal), trianglular, and rhomboid shapes, the surface area of the end surface decreases in turn, and the tip angle of the end surface becomes smaller, and thus the charge concentration increases.

**Figure 8:**
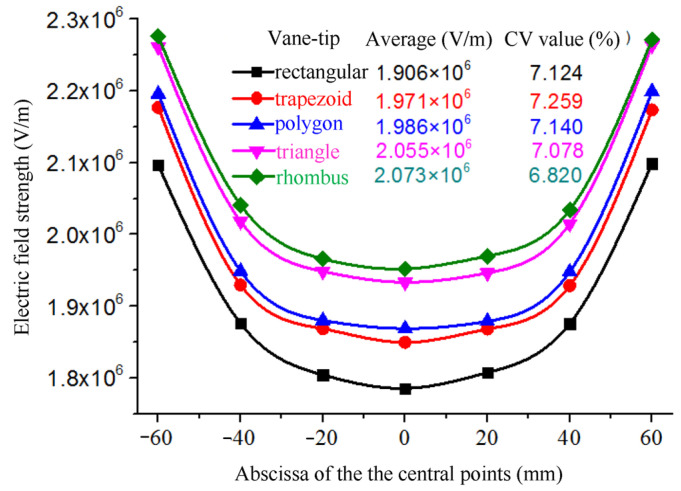
The field strength and distribution curve of the central points at the spinning-end surface of the seven nozzles with different tip shapes.

In addition, the average electric field strength and CV values of the central points of the spinning-end surface of each electrospinning system are shown in Figure 8. The average field intensity of the center points of the seven radial nozzles with rectangular tips is 1.906 × 10^6^ V/m, and the CV value of the field strength is 7.124%. From rectangular tips to trapezoidal, polygonal, triangular and rhomboid tip radial nozzles, the average field strengths of the center points increase in turn. That is, the average field strength of the radial nozzles with rhomboid tips is the largest, which is 2.073 × 10^6^ V/m, and the CV value is 6.820%, which is the smallest of all types. Therefore, the tip shape of the radial nozzle is designed to be rhomboid in this paper.

#### 2.2.4. Effects of the Tip-Pointing Direction of the Vanes on the Electric Field

In this section, the angle between the tips of the vanes and the center axis of the nozzle is taken as the research object. The downward vertical direction is taken as the reference direction. The tip pointing away from the reference direction is recorded as the “outward tip”, the tip pointing towards the reference direction is recorded as the “inward tip”, and the tip pointing parallel to the reference direction is recorded as the “upright tip”.

**Figure 9:**
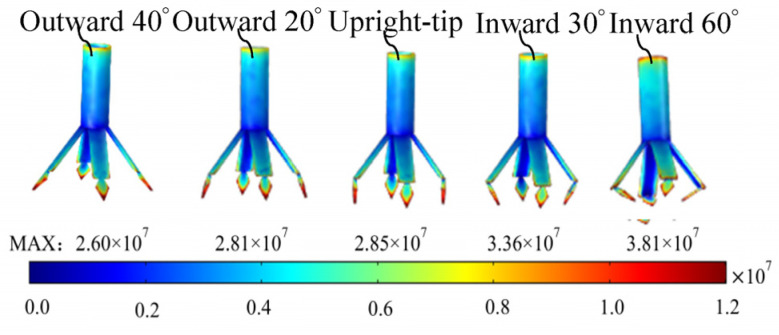
The electric field simulation of the electrospinning system models of seven radial nozzles with different tip-pointing angles.

The models of the radial nozzles with outward 40°, outward 20°, the upright tip, and inward 60° were designed and their electrostatic fields were simulated with COMSOL Multiphysics 6.0 software. As shown in Figure 9, the field intensity nephogram of the intermediate nozzles and the maximum field strengths of the electrospinning systems are depicted.

From Figure 9, it can be seen that the maximum field strength of the electrospinning systems increases gradually from 2.60 × 10^7^ V/m to 3.81 × 10^7^ V/m and increases by 46.54% as the rhomboid tips of the vanes shrink inward. This is because when the pointing angle of the tips changes to the inward direction, the electric field interference among the nozzles decreases relatively. At the same time, the angle between the tip and the vane becomes smaller, so the charges are easier to accumulate near the angles and the tips by making the electric field strength larger and larger.

**Figure 10:**
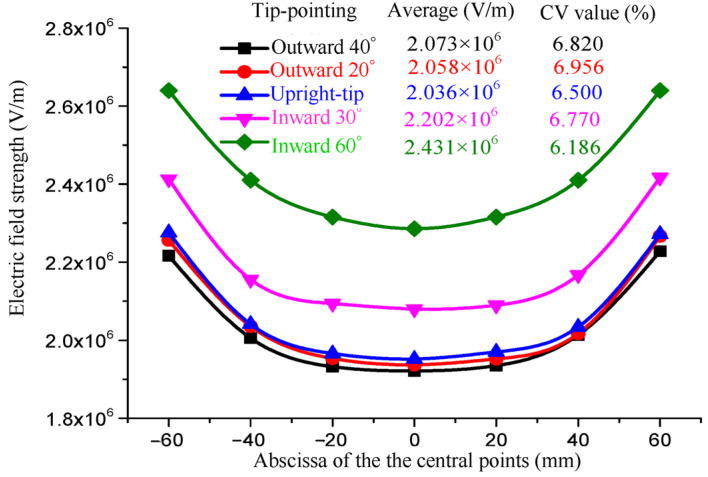
The field strength and distribution curve of the central points at the spinning-end surface of the seven nozzles with different tip-pointing angles.

In addition, the average field strength and CV values of the central points of the spinning-end surface of each electrospinning system are shown in Figure 10.

As can be seen from Figure 10, with the rhomboid tips of the vanes shrinking inward, the average field strength gradually increases from 2.073 × 10^6^ V/m to 2.431 × 10^6^ V/m and increases by 17.27%. The CV value of the electric field strength tends to decrease gradually. However, if the rhomboid tips continue to bend inward, they will affect the normal spreading of the spinning liquid in the radial nozzle. Therefore, the rhomboid radial nozzle with 60° inward tips is selected at last.

#### 2.2.5. Effect of Relative Orientation of the Vanes between the Neighboring Radial Nozzles on the Electric Field

To ensure uniform field strength across radial nozzles and minimize interference between their electric fields, when multiple nozzles are positioned at a certain distance from each other’s center axes, the average distance between the points of the high field strength needs to be determined. Consequently, by adjusting the relative positions of the vanes to their maximum extent, the average distance between the vane tips can be modified. This adjustment aims to reduce the repulsion effect caused by electric fields and mitigate end effects.

**Figure 11:**
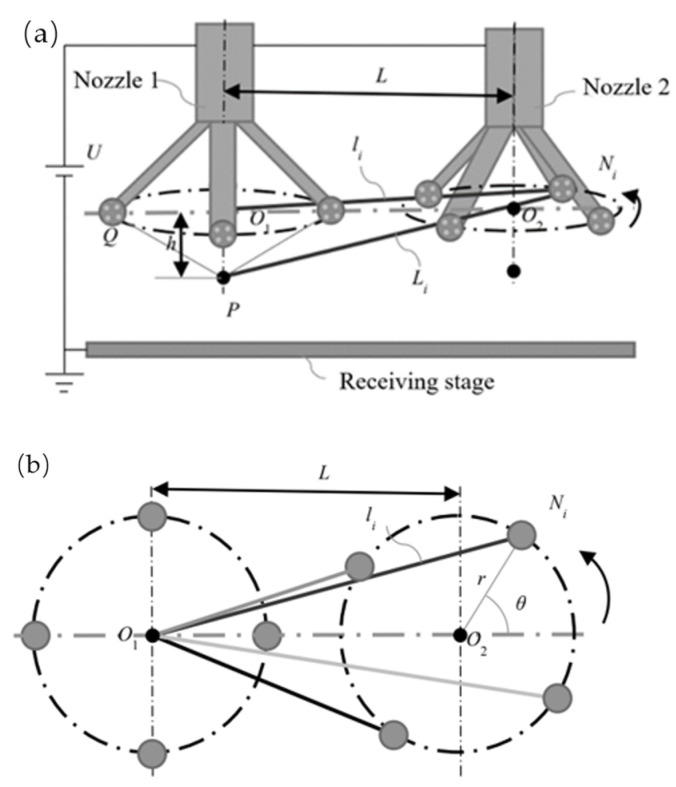
Orientation change schematic diagram of the vanes in two radial nozzles. (**a**) Axonometric view. (**b**) Vertical view.

Assuming a voltage *U* is applied to each radial nozzle with N blades (*N* ≥ 2), where all blades have identical shapes and opening angles with charge mainly concentrated at their tips, each blade can be approximated as a point charge, *Q*. If *N* point charges are uniformly distributed on a circle with radius *r*, then the angle between adjacent point charges is 2π⁄*N*. Figure 11 illustrates two nozzles denoted by *L* as the distance between them; *O*_1_ and *O*_2_ represent the centers of the circles in which the nozzle tips are located. This study focuses on investigating how variations in nozzle blade orientation affect the magnitude of the electric field strength at any given point *P* along nozzle 1’s central axis.

The electric field strength of the point charge system at the tip of the nozzle 2 blade at point *P* is calculated as:

(1) $$E=\sum_{i=1}^{N}E_{i}=\frac{1}{4\pi \varepsilon_{0}}\sum_{i=1}^{N}\frac{Q}{{L_{i}}^{2}}{\overset{⌢}{L}}_{i}$$

where *ε_0_* is the vacuum dielectric constant, *i* is the serial number of each tip in the counterclockwise direction starting from the positive direction of the x-axis, and ${\overset{⌢}{L}}_{i}$ is the unit vector pointing from the point charge *N_i_* to the target point *P*.

Based on the symmetry of the electric field, it can be observed that the orientation change in nozzle 1’s blade has no impact on the electric field intensity at point *P.* Therefore, only the influence of each blade’s charge from the adjacent nozzle, i.e., nozzle 2, on the electric field intensity at point *P* needs to be considered. Referring to Equation (1), it is evident from Figure 11 that for a given amount of charge, the strength of an electric field at a specific point solely depends on its distance.

(2) $$L_{i}=\sqrt{{l_{i}}^{2}+h^{2}}$$

(3) $$l_{i}=\sqrt{{\left[L+r\cos\left(\theta +\left(i-1\right)\cdot \frac{2\pi }{N}\right)\right]}^{2}+{\left[r\sin\left(\theta +\left(i-1\right)\cdot \frac{2\pi }{N}\right)\right]}^{2}}=\sqrt{L^{2}+r^{2}+2Lr\cos\left(\theta +\left(i-1\right)\cdot \frac{2\pi }{N}\right)}$$

Substituting Equation (3) into Equation (2) and then into Equation (1), we obtain

(4) $$E=\frac{1}{4\pi \varepsilon_{0}}\sum_{i=1}^{N}\frac{Q\cdot {\overset{⌢}{L}}_{i}}{L^{2}+r^{2}+h^{2}+2Lr\cos\left[\theta +\left(i-1\right)\cdot \frac{2\pi }{N}\right]}$$

$L^{2}+r^{2}+h^{2}$ and 2Lr all are constants, and we denote them as *C*_1_ and *C*_2_, respectively. Then, Equation (4) becomes Equation (5).

(5) $$E=\frac{1}{4\pi \varepsilon_{0}}\sum_{i=1}^{N}\frac{Q\cdot {\overset{⌢}{L}}_{i}}{C_{1}+C_{2}\cos\left[\theta +\left(i-1\right)\cdot \frac{2\pi }{N}\right]}$$

Then, for a four-blade radial nozzle, the center point of a nozzle is in the electric field of the adjacent nozzle with a field strength *E*:

(6) $$E=\frac{1}{4\pi \varepsilon_{0}}\left[\frac{Q}{C_{1}+C_{2}\cos\theta }+\frac{Q}{C_{1}-C_{2}\sin\theta }+\frac{Q}{C_{1}-C_{2}\cos\theta }+\frac{Q}{C_{1}+C_{2}\sin\theta }\right]=\frac{\left(4{C_{1}}^{3}-2C_{1}{C_{2}}^{2}\right)Q}{4\pi \varepsilon_{0}\left(-{C_{2}}^{4}\cos^{4}\theta +{C_{2}}^{4}\cos^{2}\theta +{C_{1}}^{4}-{C_{1}}^{2}{C_{2}}^{2}\right)}$$

Make $x=\cos^{2}\theta$, and then Equation (6) can be simplified as

(7) $$E=\frac{1}{4\pi \varepsilon_{0}}\cdot \frac{\left(4{C_{1}}^{3}-2C_{1}{C_{2}}^{2}\right)Q}{-{C_{2}}^{4}x^{2}+{C_{2}}^{4}x+{C_{1}}^{4}-{C_{1}}^{2}{C_{2}}^{2}}$$

Let a function $f\left(x\right)=-{C_{2}}^{4}x^{2}+{C_{2}}^{4}x+{C_{1}}^{4}-{C_{1}}^{2}{C_{2}}^{2}$, derivative of both sides with respect to *x*, and $f^{\prime }\left(x\right)=0$ obtains: equal x=1/2: when θ=π/4, f(x) takes maximum value, and in this case, the electric field intensity E should be minimized. According to the “edge effect” in the electrospinning process, each nozzle corresponds to its own jet, and the electric field generated by other nozzles exerts a Coulomb repulsive force on it. Therefore, minimizing the electric field force from other nozzles is desirable. In other words, when the field strength generated by other nozzles is minimized, the weakening of the current nozzle’s field strength is also minimized. For a four-blade radial nozzle with blades defined as “reference azimuths” in a cross-shaped arrangement, if we consider the rotation angle of a blade deviating counterclockwise from this reference azimuth as the rotation angle, then when this rotation angle is 45°, the electric field force on the central axis of the current nozzle experiences minimal weakening. Consequently, achieving more uniform field strengths among all nozzles can be attained.

**Figure 12:**
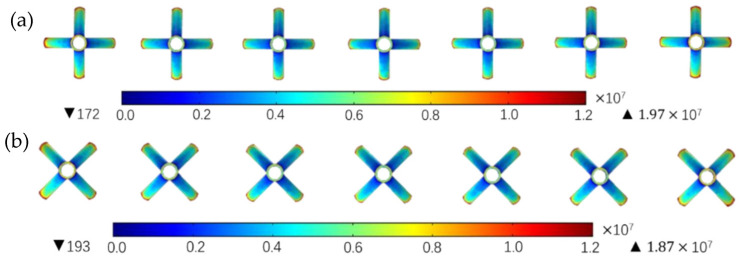
Electric field nephogram of the radial nozzles with rectangular vanes in different positions—vertical view. (**a**) Reference azimuth; (**b**) 45° rotation azimuth.

As shown in Figure 12, the electrostatic field simulation of an ordinary rectangular-blade radial seven-nozzle electrospinning system with two limit positions was carried out.

It can be seen that the peak field strength in the seven-nozzle electrostatic spinning system is 1.97 × 10^7^ V/m when aligned in the reference orientation, and the peak field strength is 1.87 × 10^7^ V/m when the adjacent nozzle blades are aligned at an angle of 45°, which is smaller than the former. This is in agreement with theoretical calculations for a uniform electric field in the blade row orientation.

**Figure 13:**
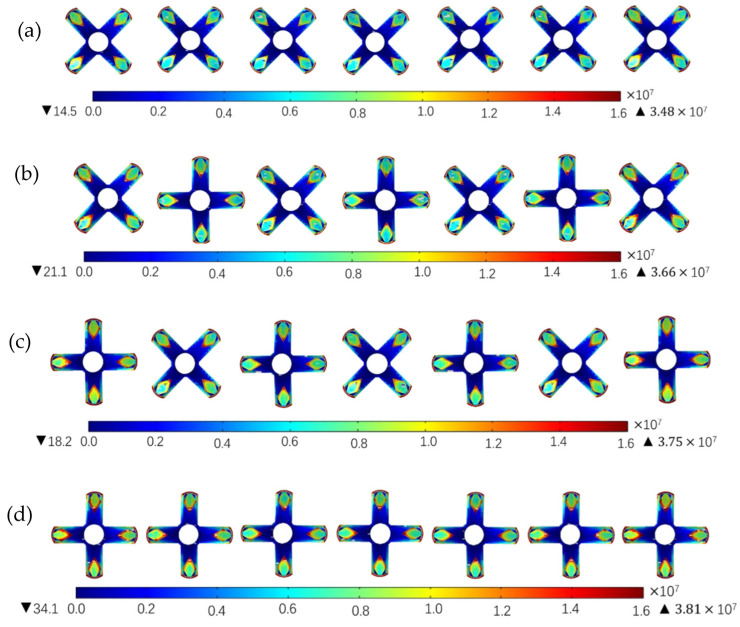
Electric field nephogram of the radial nozzles with diamond tips in different positions. (**a**) All nozzles are arranged in 45° rotation azimuth. (**b**) The reference azimuth and 45° rotation azimuth are arranged alternately—the center nozzle is the reference azimuth. (**c**) The reference azimuth and 45° rotation azimuth are arranged alternately—the center nozzle is 45° rotation. (**d**) Arranged according to the reference azimuth.

In order to balance the magnitude of the field strength and the CV value of the field strength, the radiating nozzles pointing inward at an angle of 60° from the diamond-shaped tip of the previous study were arranged in a reasonable manner according to the reference orientation or the 45° rotational orientation. And the electric field simulation was carried out as shown in Figure 13; four scenarios were simulated:

(a) All nozzles are arranged in a 45° rotation and the peak field strength in this model is 3.48 × 10^7^ V/m;

(b) Nozzles are alternated in the reference orientation and 45° rotational orientation and the central nozzle is the reference orientation nozzle; the peak field strength in this model is 3.66 × 10^7^ V/m;

(c) Nozzles are arranged alternately in the reference orientation and 45° rotational orientation and the central nozzle is the 45° rotational orientation nozzle; the peak field strength in this model is 3.75 × 10^7^ V/m;

(d) All nozzles are arranged in the reference orientation and the peak field strength in this model is 3.81 × 10^7^ V/m.

The four schemes are illustrated in Figure 13. The number of nozzles oriented at 45 degrees is (a) 7, (b) 4, (c) 3, (d) 0. Correspondingly, the peak field strength also increases sequentially. This observed pattern confirms the theoretical influence of the radial nozzle blade layout orientation on the electric field.

**Figure 14:**
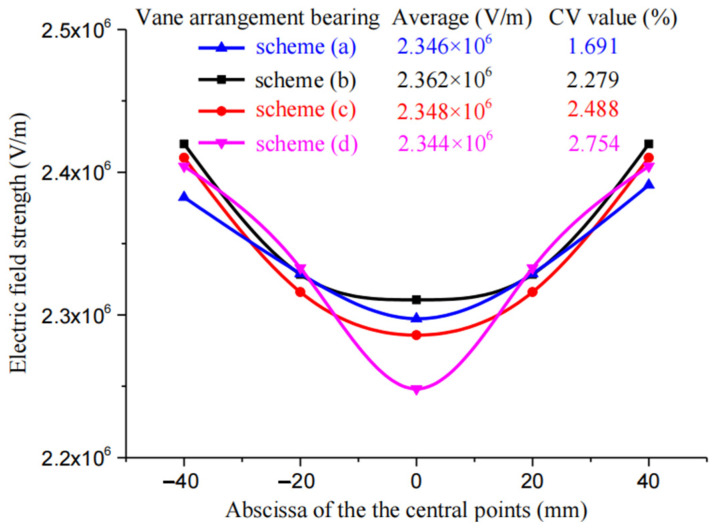
Comparison of electric field intensity of the nozzles with inward 60° diamond tips arranged in different positions.

In multi-nozzle electrospinning, due to the fact that only one side of each end nozzle is exposed to an electric field relative to the other nozzles, there exists a greater imbalance in the electric field distribution. Consequently, in electrospinning experiments, the two end nozzles are generally not utilized for spinning purposes but rather serve as balancing elements for maintaining uniformity within the middle nozzle’s electric field. Therefore, Figure 14 presents (a) through (d), where CV values at filament endpoints sprayed by five nozzles positioned at different locations determine the uniformity of their respective electric fields.

Figure 14 shows four schemes; in the middle position of the five nozzles in the middle of the filament end face field strength average value, the is basically the same, about 2.350 × 10^6^ V/m. From scheme (a) to scheme (d), the field strength CV value increases from 1.691% to 2.754%, indicating that the scheme (a) in the middle of the five nozzles of the electric field uniformity is better. Therefore, this paper finally determines the linearly arranged radial multi-nozzle blade arrangement as all the nozzles being arranged in the 45° rotational orientation.

## 3. Materials and Experiments

### 3.1. Materials and the Solution

Polyacrylonitrile (PAN, *M_w_* = 85,000) was purchased from Shanghai Aladdin Bio-Chem Technology Co., Ltd., Shanghai, China. N, N-dimethylformamide (DMF) was supplied by Tianjin Kemiou Chemical Reagent Co., Ltd., Tianjin, China. TiO_2_ (P25, 20 nm) was provided by Shanghai Hansi Chemical Co., Ltd., Shanghai, China.

PAN solution (10% by wt.) was prepared by dissolving the PAN solute in the solvent of DMF (1:9 by wt.) and mixing by magnetic stirring for 4 h at room temperature to obtain homogenous solution. TiO_2_@PAN solution was prepared by blending TiO_2_ nanoparticles into the previously prepared 10% PAN solution at the ratios of 1.78% by wt., with the aid of magnetic stirring for 2 h at 70 °C.

### 3.2. Electrospinning Experiment

**Figure 15:**
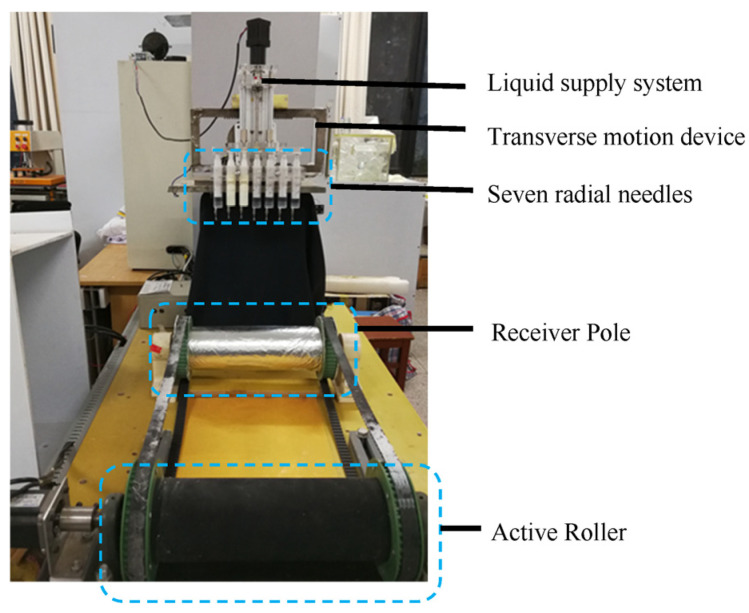
The in-house electrospinning device with seven radial nozzles.

The spinning emitter in the experimental device adopts the optimal structure of seven radial nozzles discussed earlier: four vanes; the length of vanes is 6 mm; rhomboid tips, 60° inward; and all the nozzles are arranged in the 45° rotational orientation. The electrospinning experiment was performed by the in-house device with seven optimal radial nozzles, as shown Figure 15, and the spinning solution was the TiO_2_@PAN solution prepared in the previous section. Table 2 shows the types and sources of the related instruments.

**Figure 16:**
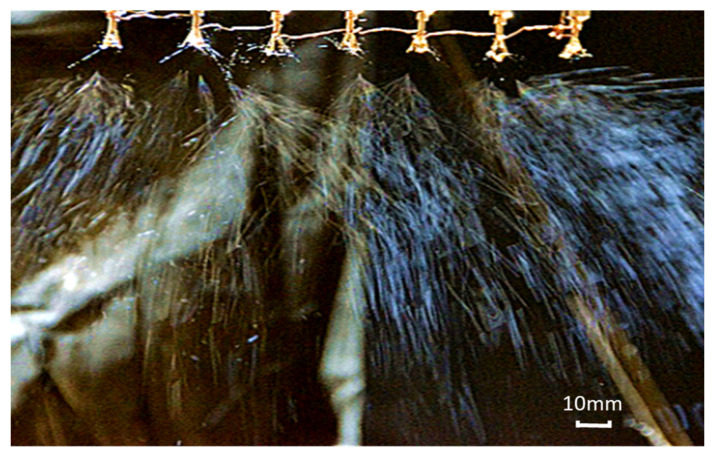
Electrospinning experiment with seven radial nozzles.

The feeding rate was 2.67 mL/h. The receiving distance was 200 mm. The DC power of 20 kV was supplied by the positive voltage to the radial nozzle, and a negative voltage of 3 kV was applied to the receiving electrode. The spinning time was 15 min. The spinning process is shown in Figure 16.

## 4. Results and Discussion

**Figure 17:**
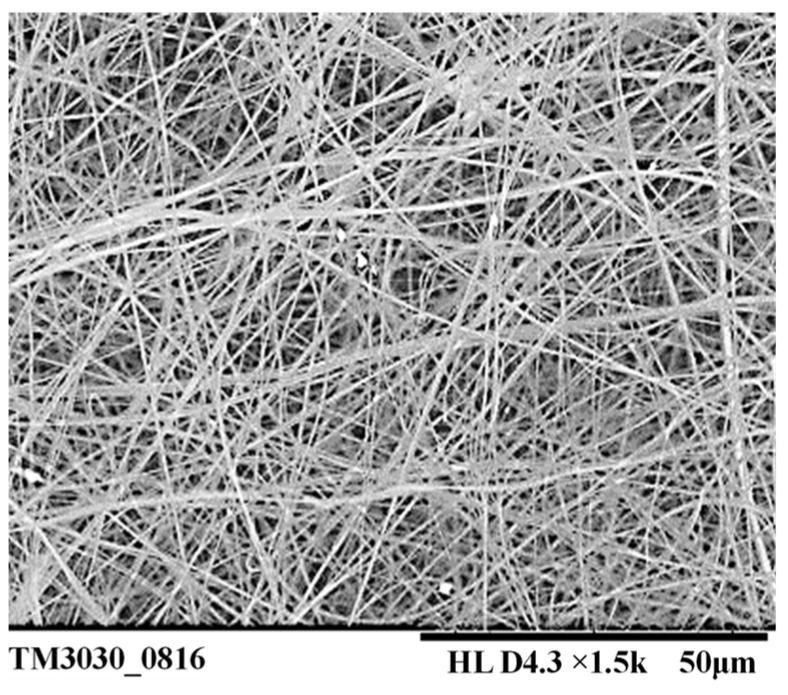
SEM of the prepared fibers.

**Figure 18:**
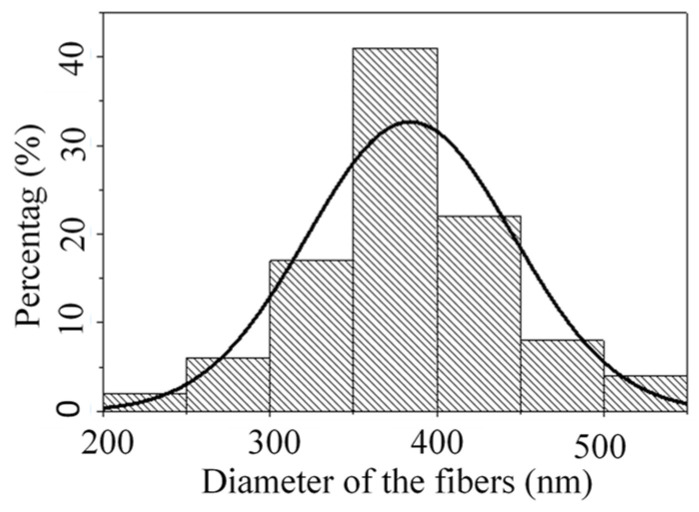
The diameter distribution of the prepared fibers.

As can be seen from Figure 16, each radial nozzle can produce multiple jets in the electrospinning process. Therefore, it is advantageous to increase the output of the nanofibers. The scanning electron microscopy (SEM) image of the nanofiber film prepared in the electrospinning experiment is shown in Figure 17. And then the diameters of one hundred fibers were measured with the software named Image-Pro Plus6.0, and the diameter distribution is shown in Figure 18. Finally, the average diameter and CV value of the fibers were calculated, and they were 384.11 nm and 16.06%, respectively.

Five 5 cm × 5 cm samples were cut down along the transverse direction of the fiber membrane produced, and their thicknesses were measured with the film-thickness-measuring instrument whose type is CHY-C2. Twenty measuring points were randomly selected from each sample to calculate the average value. The data are listed in Table 3. The average thickness of the total fiber membrane is 0.1167 mm. At the same time, the CV value of the transverse thickness of the prepared fiber membrane was calculated and it is 8.69%.

In addition, the porosity of the prepared fiber membrane was calculated. A piece of 2 cm × 2 cm fiber membrane was cut down from the fourth sample, whose fiber film thickness is close to the average thickness. The thickness was measured again and *h* = 0.1196 mm was obtained. Then, we used an electronic balance to weigh its mass, *M*, and it was 0.0054 g. The porosity of the fiber membrane can be calculated with Formulas (8) and (9).

(8) $$\rho_{1}=\frac{M\cdot 10}{h\cdot S}$$

(9) $$\eta =(1-\frac{\rho_{1}}{\rho_{2}})\times 100\%$$

Among them, $\rho_{1}$($g/{\mathrm{cm}}^{3}$), *M* (g), *h* (cm), *S* (cm^2^), and *η* are the apparent density, mass, thickness, area, and porosity of the fiber membrane, respectively, and $\rho_{2}$($g/{\mathrm{cm}}^{3}$) is the density of the raw materials.

According to Formula (8), the apparent density of the fiber membrane was calculated, and it is $\rho_{1}=0.1129g/{\mathrm{cm}}^{3}$. Then, the porosity according to Formula (9) was calculated and we obtained η=91.31%. It can be seen that the electrospun membranes prepared with the radial nozzles studied in this paper have high porosity.

## 5. Conclusions

In this paper, COMSOL Multiphysics 6.0 software was used to simulate the electric field of the electrospinning with seven radial nozzles, and the influence on the electric field intensity and distribution of the structural parameters of the radial nozzle, including the number, length, tip-shape, and tip-pointing direction of the vanes, were studied. Through simulation studies, the optimum structure parameters of the radial nozzle were obtained: the number of the vanes is four; the vane length is 6 mm; the tip shape of the radial nozzle is designed to be rhomboid; and the rhomboid radial nozzle with 60° inward tips is selected at last. And in order to improve the uniformity of the electric field in the electrospinning of linearly arranged multi-gap radial nozzles, this paper obtains the electric field strength at any point on the central axis of a given radial nozzle based on the principle of electric field superposition and then derives the blade rotation angle corresponding to the minimum Coulomb repulsion force at the target point. Using COMSOL Multiphysics 6.0 software to simulate the electric field of the seven-nozzle linear model with different blade orientations, the correctness of the uniform electric field theory with the blade orientation is verified. It is found that the 45° rotational orientation of the adjacent nozzle blades is conducive to the uniformity of the radial multi-nozzle electric field, and the CV value of the field strength of seven-nozzle electrospinning can reach 1.691% under this arrangement. Then, electrospinning with seven radial nozzles was performed, and it was found that each radial nozzle can produce multiple jets in the electrospinning. And the average diameter of the prepared electrospun membrane is 384.11 nm, and the CV value is 16.06%. In addition, the CV value of the transverse thickness and the porosity of the prepared fiber membrane is 8.69% and 91.31% respectively. It can be seen that the electrospun membranes prepared with the radial nozzles studied in this paper have even thickness and high porosity; what is more, the fibers are relatively finer and more uniform.

## Figures and Tables

**Table 1:** Parameters of the electrospinning system.

*l* (mm)	*h* (mm)	Receiver *L_r_* × *W_r_* × *H_r_* (mm)	Air *L_a_* × *W_a_* × *H_a_* (mm)
20	200	500 × 400 × 1	650 × 650 × 400

**Table 2:** Experimental instruments.

Instruments	Type	Source
Spinning device	Seven radial nozzles	In-house
DC high-voltage power supply	DW-P/N603	Tianjin Dongwen High Voltage Power Supply, Ltd., Tianjin, China
Metal halide lamp	70W	Xincheng Lighting, Ltd., Huzhou, China
Motor agitator	DF-101S	Gongyi Yuhua Instrument Co., Ltd., Zhengzhou, China
Thermostat water bath	HH-4	Kexi Instrument, Ltd., Changzhou, China
Camera	Sony DSC-TX9	Sony, Beijing, China
Electron microscope	TM3030	Techcomp (China) Ltd., Beijing, China
Film-thickness-measuring instrument	CHY-C2	Jinan Labthink Mechatronics Technology Co., Ltd., Jinan, China

**Table 3:** Thickness of the samples on the fiber membrane.

Sample	1	2	3	4	5
Thickness (mm)	0.1046	0.1072	0.1261	0.1205	0.1252

## Data Availability

Data is contained within the article.
